# Role of Armadillo repeat 2 and kinesin-II motor subunit Klp64D for wingless signaling in *Drosophila*

**DOI:** 10.1038/s41598-020-70759-8

**Published:** 2020-08-17

**Authors:** Linh Thuong Vuong, Jong-Hoon Won, Minh Binh Nguyen, Kwang-Wook Choi

**Affiliations:** 1 Department of Biological Sciences, Korea Advanced Institute of Science and Technology, Daejeon, 34141 Korea; 2 Present Address: Department of Cell, Developmental and Regenerative Biology, Icahn School of Medicine at Mount Sinai, New York, NY 10029 USA

**Keywords:** Cell biology, Developmental biology

## Abstract

Armadillo (Arm) is crucial for transducing Wingless (Wg) signaling. Previously, we have shown that Klp64D, a motor subunit of *Drosophila* kinesin-II, interacts with Arm for Wg signaling. Molecular basis for this interaction has remained unknown. Here we identify a critical Arm repeat (AR) required for binding Klp64D and Wg signaling. Arm/${\varvec{\beta}}$-catenin family proteins contain a conserved domain of 12 Arm repeats (ARs). Five of these ARs can interact with Klp64D, but only the second AR (AR2) binds to the cargo/tail domain of Klp64D. Overexpression of AR2 in wing imaginal disc is sufficient to cause notched wing margin. This phenotype by AR2 is enhanced or suppressed by reducing or increasing Klp64D expression, respectively. AR2 overexpression inhibits Wg signaling activity in TopFlash assay, consistent with its dominant-negative effects on Klp64D-dependent Wg signaling. Overexpression of the Klp64D cargo domain also results in dominant-negative wing notching. Genetic rescue data indicate that both AR2 and Klp64D cargo regions are required for the function of Arm and Klp64D, respectively. AR2 overexpression leads to an accumulation of Arm with GM130 Golgi marker in Klp64D knockdown. This study suggests that Wg signaling for wing development is regulated by specific interaction between AR2 and the cargo domain of Klp64D.

## Introduction

The Wnt signaling pathway controls diverse biological processes such as growth, patterning and differentiation. Misregulation of the Wnt pathway can lead to human diseases including cancer and metabolic disorders^1–3^. Wnt signaling is evolutionarily conserved, and hence *Drosophila* has been extensively utilized for genetic dissection of the Wnt signal transduction pathway. Secreted Wingless (Wg, *Drosophila* Wnt1) activates a cascade of signal transduction events. Armadillo (Arm, β-catenin homolog) plays a key role in transducing the canonical Wnt/Wg signaling by acting as a transcriptional regulator^4^. Arm is also an essential component of adherens junctions (AJs) together with E-cadherins (E-cad) and $\beta$-catenin in epithelial cells^5,6^. In the absence of Wg signal, Arm is accumulated at the adherens junctions (AJs) where it forms a complex with E-cad or is rapidly degraded ^7^. Expression of Wg target genes is activated when stabilized Arm enters the nucleus and recruits other co-activators^8^.

Arm is required for development of fly organs at different stages. It has been shown that loss of Klp64D causes mislocalization of Arm in differentiating photoreceptor cells of pupal retina^9^. Klp64D, the *Drosophila* homolog of Kif3A, is a subunit of the kinesin-II microtubule-based heterotrimeric motor protein complex^10^. Recently, we have reported that Klp64D is also required for Wg signaling as a binding partner of Arm^11^. Klp64D recruits Arm and its interacting partner Dishevelled (Dsh) for Wg signal transduction. In wing development, Wg signaling is induced from the dorso-ventral (DV) boundary of wing imaginal disc. In the absence of kinesin-II function, Arm is abnormally accumulated with intracellular vesicles in the DV boundary region. Thus, it has been suggested that Klp64D might be involved in intracellular trafficking of Arm for Wg signaling^11^. However, specific regions of Arm and Klp64D proteins that are critical for their binding and Wg signaling have not been determined.

It has been shown that distinct roles of Arm at AJs and Wg signaling are genetically separable. The N-terminal region of Arm plays a role in AJs while the C-terminal domain is essential for Wg signaling function^12^. Arm contains a long repeat region called the Arm repeat (AR) domain in the middle portion of the protein. Arm repeats (ARs) are evolutionarily conserved structures found in many proteins^13,14^. Metazoan AR-containing proteins have different numbers of AR units, ranging from 1 (ARMC1) to 13 (ARMC4)^15^. The AR regions of Arm and mammalian $\beta$-catenin consist of 12 repeats. Each AR consisting of about 40 amino acid residues has a characteristic structure with three alpha-helices. The AR repeats of $\beta$-catenin form a concave groove region called an ARM domain that binds competitively to cadherins, APC and Tcf^14,16–18^. APC and Tcf compete for binding Arm because the ARM domain is the main binding site that typically interacts with one partner of the destructive complex at a time^19,20^. In contrast, the BCL9 homolog Legless (Lgs), which also interacts with Arm for Wg target gene expression, can bind to a different region of Arm repeats without competition with Tcf^21–23^. Thus, although the central groove region of Arm is a major site for binding to partners, other individual Arm repeats may also be involved in interaction with partners. In our earlier study, the AR region of Arm was shown to interact with the coiled-coil domain and/or the C-terminal cargo domain of Klp64D^11^. It is an intriguing question whether Klp64D interacts with a specific AR region(s) to regulate Wg signaling.

In this study, we analyzed the interactions between different ARs of Arm and Klp64D kinesin-II motor subunit. We demonstrate that AR2 specifically interacts with the cargo domain of Klp64D. Our data indicate that AR2 and the Klp64D cargo domain are essential for the function of Arm and Klp64D in vivo, respectively. Overexpression of these interacting domains are sufficient to antagonize Wnt signaling. This study suggests a role of AR2 and Klp64D cargo domain in Wg signaling and provides an insight into a new tool for inhibiting Wnt signaling through dominant-negative interactions.

## Results

### Identification of Arm repeat binding to Klp64D

**Figure 1:**
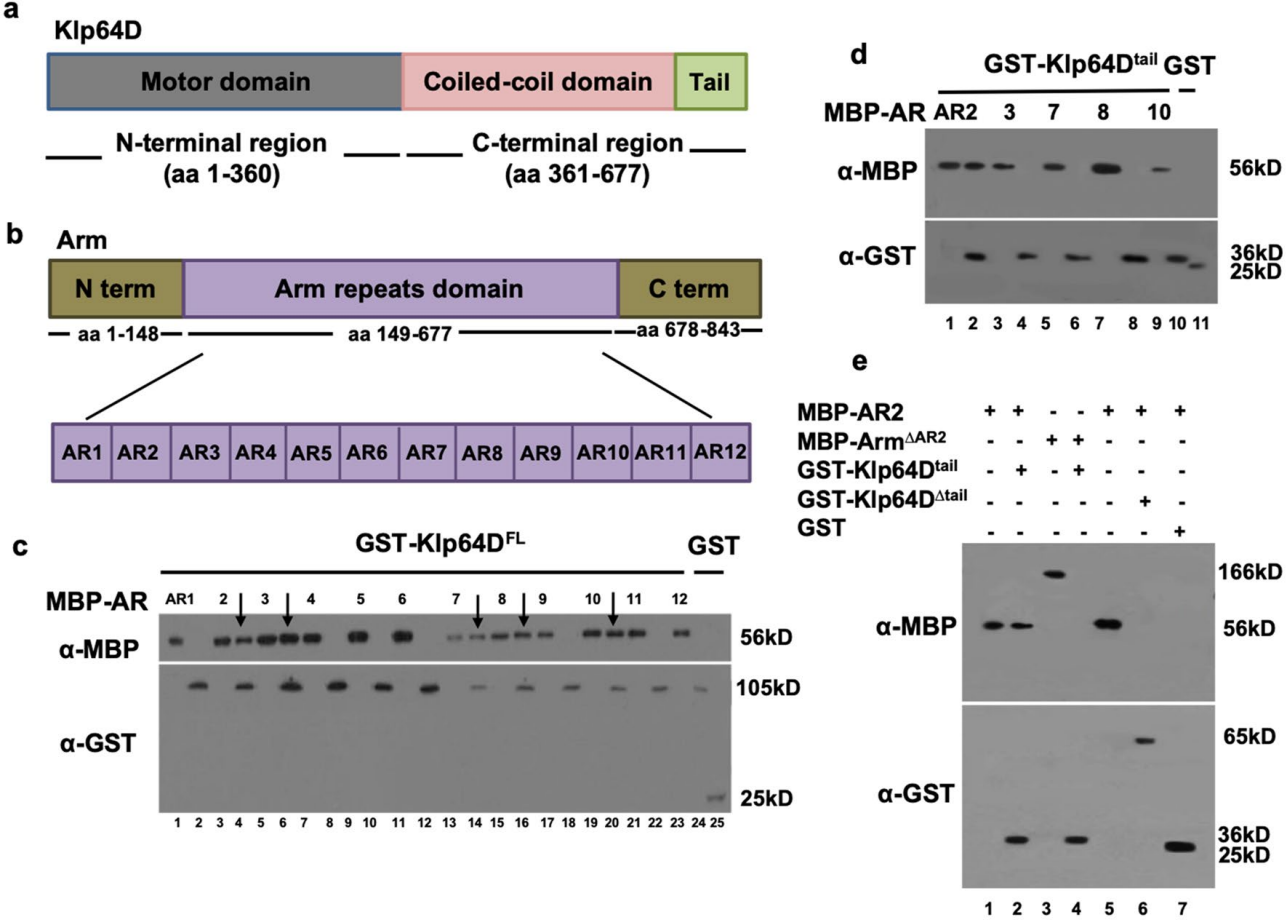
Klp64D tail domain and AR2 show physical interaction. **(a)** The domain structure of *Drosophila* kinesin-II. The N-terminal region contains the catalytic motor domain or head domain (aa1–360). The C-terminal region consists of a coiled coil stalk domain (aa 361–581) and the tail domain (aa 582–677). **(b)** The domain structure of Arm. Twelve Arm repeats are labeled as AR1-AR12. Each domain consists of about 40 amino acid residues. **(c)** Twelve individual AR domains of Arm were pulled down by GST-Klp64D^FL^ (Full-length Klp64D) (even number lanes) or GST (lane 25). Odd number lanes are 10% input for MBP-AR1 through AR12, respectively). **(d)** Five AR domains of Arm (AR2, 3, 7, 8, 10) (lanes 1, 3, 5, 7, 9 are 10% input) were pulled down by GST-Klp64D^tail^ (Klp64D^T^) (lanes 2, 4, 6, 8 and 10) (arrows) or GST (lane 11). **(e)** Klp64D^tail^ and AR2 are necessary and sufficient for binding between Klp64D and Arm. MBP-AR2 shows direct binding with GST-Klp64D^tail^ (lane 2). Arm^ΔAR2^ or Klp64D^Δtail^ does not bind to Klp64D^tail^ or AR2, respectively (lanes 4, 6). Input 10% (lanes 1, 3, 5) and GST (lane 7). Western blots in (c-d) are representative results from three experiments.

Previously, we showed that Klp64D and Arm are associated in a protein complex in which Arm repeat domains directly interact with the C-terminal region of Klp64D^11^. The N-terminal region of Klp64D contains the motor domain whereas the C-terminal region consists of a coiled-coil domain and the tail domain for cargo binding (Fig. 1a). Arm protein contains a conserved middle region that consists of 12 repeats of similar Arm repeat (AR) domains (labeled as AR1–AR12) (Fig. 1b). To identify the specific AR region of Arm involved in the physical interaction with Klp64D, we tested binding between GST-Klp64D and each of 12 MBP-AR fragments. Five of these twelve Arm repeats, AR2, 3, 7, 8 and 10, were pulled down by Klp64D (Fig. 1c, Fig. S1a).

Arm can bind to the C-terminal half of Klp64D, implying its interaction with the coiled-coil domain and/or the C-terminal cargo domain^11^. Interestingly, GST-pulldown assays showed that only AR2 among these five Arm repeats could bind to the tail domain of Klp64D (Klp64D^tail^) (Fig. 1d, Fig. S1b). These data suggest that AR2 is critical for interacting with the cargo domain of Klp64D. Since an individual AR peptide may not fold properly, AR2 binding to Klp64D could be due to non-specific interaction. Hence, we tested whether AR2 is necessary for binding to the tail domain of Klp64D. To test this possibility, we generated Arm mutant protein deleted in the AR2 domain (Arm^ΔAR2^) and Klp64D mutant protein deleted in the C-terminal tail domain (Klp64D^Δtail^). Pull-down analysis indicated that Arm^ΔAR2^ cannot bind the Klp64D tail while Klp64D^Δtail^ fails to bind AR2 (Fig. 1e, Fig. S1). These results demonstrate that AR2 and the tail domain of Klp64D are necessary for binding Klp64D tail and AR2, respectively. In S2 cell extract, AR2-Myc was co-immunoprecipitated with Klp64D-Flag but not with a different kinesin protein Klp61F-Flag, suggesting that AR2 forms a complex with Klp64D (Fig. S2).

### Overexpression of AR2 causes wing notching and inhibits Wg signaling

We have identified 5 AR regions that bind to Klp64D (Fig. 1b, c). To determine whether overexpression of these AR domains show any dominant effect, we generated *UAS-AR* transgenic flies. To detect the expression of these transgenes, AR constructs were designed to be expressed as AR-HA fusion proteins. We could detect the expression of AR-HA constructs for AR3, 7, 8, and 10 in wing disc, but AR2-HA expression was not detectable. Hence, we generated untagged *UAS-AR* transgenes inserted at the same chromosomal site (59D3 on 2R) using phiC31 integrase ^24^ to induce similar expression levels. Ubiquitous overexpression of AR2, 3, 7, or 8 using *act-Gal4* caused larval lethality while AR10 did not affect development. Thus, overexpression of AR2, 3, 7 and 8 can dominantly interfere with normal development.

**Figure 2:**
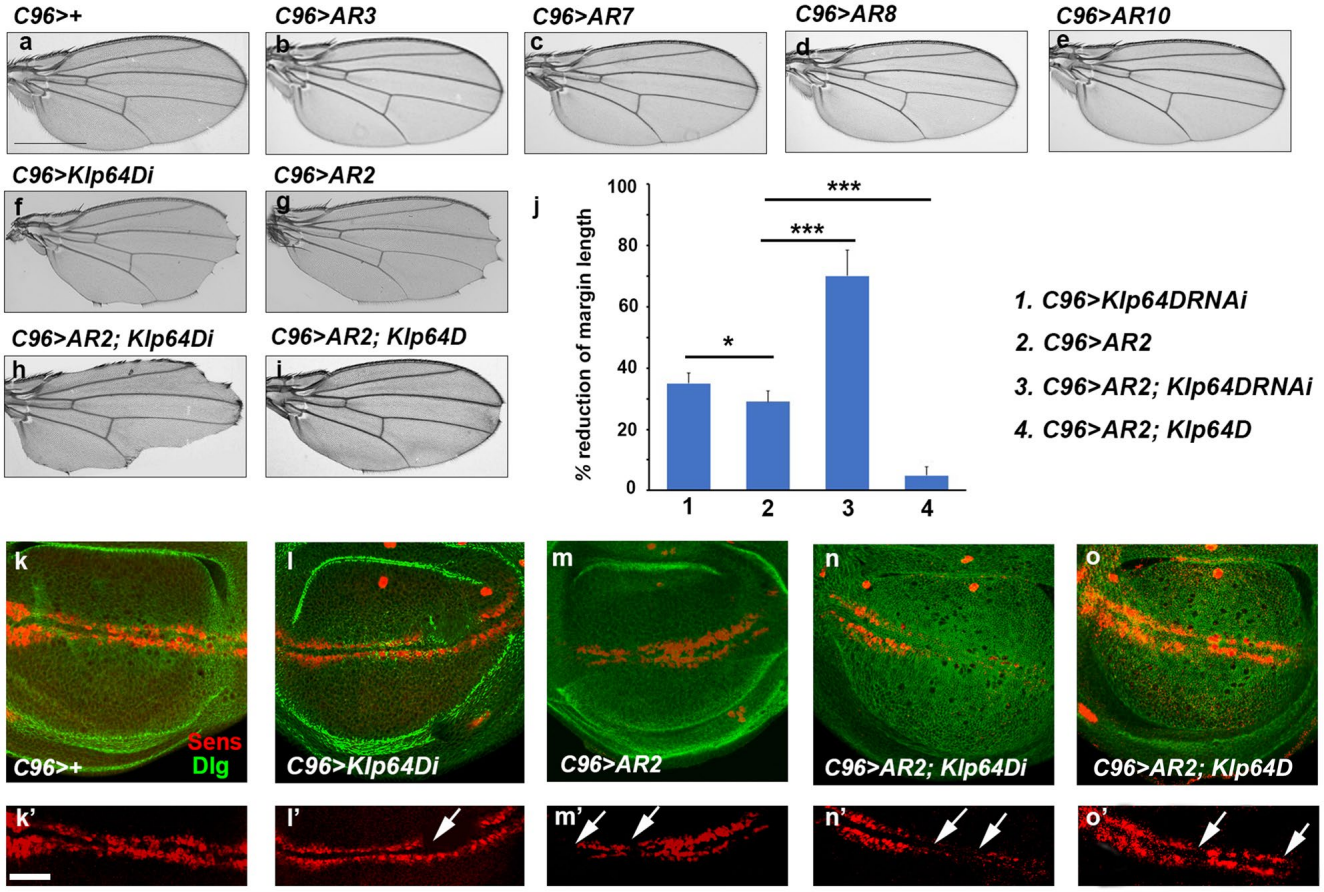
AR2 domain interferes with Klp64D function. **(a)** *C96-Gal4* >  + as a control. **(b–e)** Overexpression of repeat domains (AR3, AR7, AR8 and AR10) by *C96-Gal4* shows normal wings. **(f–i)** Genetic interaction between AR2 and Klp64D in the adult wing. **(f)** *C96* > *Klp64D RNAi* (*Klp64Di* in short) shows notched wing phenotype. **(g)** Overexpression of AR2 shows notching in the wing margin. **(h)** *C96* > *AR2; Klp64Di* shows more severe notching phenotype. **(i)** Overexpression of *Klp64D* strongly rescues the wing phenotype caused by AR2 overexpression. **(j)** Quantification of notched wing phenotypes shown in **(f–i)**. **(k–o)** Effects of AR2 and Klp64D on Sens expression. *C96* >  + control shows no effect on Sens expression **(k)**. *C96* > *Klp64Di* and *C96* > *AR2* cause partial loss of Sens **(l, m)**. *C96* > *AR2* phenotype is enhanced or suppressed by co-expression of *Klp64Di* **(n)** or Klp64D **(o)**, respectively. **(k’–o’)** Red channels of **k–o**. Arrows indicate a reduction or loss of Sens expression. The scale bar is equal to 100 µm **(a–i)** or 50 µm **(k–o’)**.

Next, to test whether overexpression of AR proteins can impair Klp64D function in vivo, each of AR transgenes was overexpressed along the DV boundary region of wing disc using *C96-Gal4*. Overexpression of AR3, 7, 8 or 10 that fails to bind the tail domain of Klp64D did not cause any effect on the wing (Fig. 2a–e). In contrast, overexpression of AR2 in the DV boundary region resulted in notching along the wing margin (n > 50 wings) (Fig. 2g). For quantification of wing notching, we measured the length of notched wing margin. AR2 overexpression resulted in 29 ± 3% reduction in the margin length (Fig. 2j). To determine if the effects of AR2 overexpression were due to its specific interference with Klp64D, we tested whether the AR2 effects can be altered by decreasing or increasing the level of Klp64D. Klp64D RNAi showed similar but slightly more notching than AR2 overexpression (35 ± 2% loss of margin, n = 50 wings) (Fig. 2f, g, j). The wing notching phenotype by AR2 overexpression was enhanced by Klp64D RNAi knockdown with 70 ± 6% reduction in wing margin (n = 50 wings) (Fig. 2h, j). Since such enhancement might be an additive effect of AR2 and Klp64D RNAi, we tested whether the phenotype of AR2 overexpression can be suppressed by increasing the level of Klp64D. While Klp64D overexpression in the wild-type background had no effect on wing development ^11^, the effects of AR2 overexpression was strongly suppressed by Klp64D overexpression (5 ± 2% loss of margin, n = 30 wings) (Fig. 2i, j). The notched wing phenotype resulting from AR2 expression might be related to cell death. Hence, we tested whether the AR2 phenotype can be suppressed by overexpressing p35 cell death inhibitor ^25^. Wing notching caused by Arm knockdown (*C96* > *arm RNAi*) was not significantly rescued by p35 overexpression (Fig. S3). While p35 efficiently suppressed cleaved caspase 3 (Cas3) staining induced by overexpressing proapoptotic gene *hid* (Fig. S4). AR2 overexpression did not induce ectopic Cas3 staining (Fig. S4), and the notched wing phenotype of AR2 overexpression was not noticeably suppressed by p35 (Fig. S3). These data suggest that wing notching caused by Arm knockdown or AR2 overexpression is unlikely due to cell death.

It has been shown that Senseless (Sens), a downstream target of Wg signaling, is reduced by Klp64D knockdown using *C96-Gal4* (Fig. 2l) ^11^ when comparing to the control (Fig. 2k). AR2 overexpression (*C96* > *AR2*) also inhibited the expression of Sens, thus showing partial disruption of Sens staining along the DV boundary (Fig. 2m). The defects in Sens expression by AR2 overexpression was considerably enhanced (Fig. 2n) or suppressed (Fig. 2o) by Klp64D RNAi or overexpression, respectively. These results suggest that a high level of AR2 specifically interferes with the interaction between Klp64D and Arm, thus acting as a dominant-negative factor.

**Figure 3:**
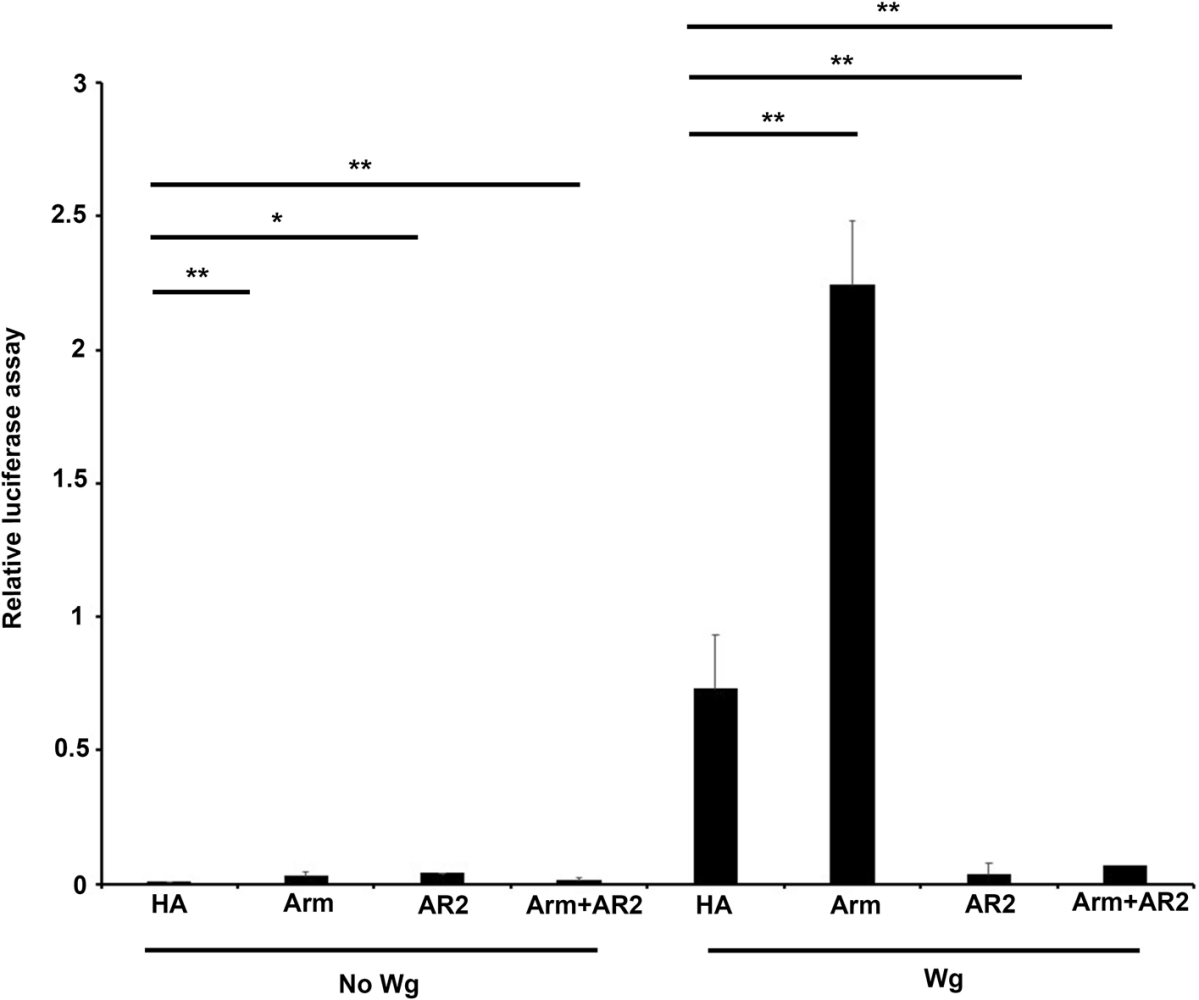
AR2 inhibits Arm function in Wg signaling. Assay for Wg signaling activity in the cells expressing control HA, Arm, AR2 or Arm plus AR2, respectively. The relative luciferase activity indicates the ratio of firefly and renilla luciferase activities. Culture media from tub-Wg S2R+ cell was added 1 day before cell lysis to induce signaling. Overexpression of Arm increases the relative luciferase activity, whereas overexpression of AR2 in the presence or absence of Arm causes a strong reduction of Wg signaling (** p < 0.001, five independent assays).

Since Klp64D is required for Wg signaling, the dominant-negative effects of AR2 in the wing margin may be related to an inhibition of Wg signaling. To determine whether AR2 can directly antagonize Wg signaling, we carried out TopFlash assays for Wg signaling activity by measuring the ratio of Tcf-responsive firefly luciferase reporter and constitutive Renilla luciferase reporter expression. S2R + cells transfected with *UAS-HA* were used as control in the absence or presence of exogenous Wg secreted in the medium. Wg-containing medium was added 4 days after transfection to induce the downstream signaling. In the absence of Wg, the luciferase ratio was near zero in the presence or absence of exogenous Arm (Fig. 3). An addition of Wg increased the relative luciferase activity to 0.7 ± 0.3 from the background level near zero, indicating Wg signaling in the absence of exogenous Arm. This activity was further increased to approximately 2.3 ± 0.3 by expressing Arm. AR2 overexpression had little effect on the viability of culture cells (Fig. S5) as in wing discs (Fig. S4). In the absence of exogenous Arm, AR2 inhibited relative luciferase activity nearly to the basal level (0.03 ± 0.02). Overexpressing AR2 together with Arm also decreased Wg signaling nearly to the basal level (0.06 ± 0.04) (Fig. 3). These results suggest that overexpression of AR2 repeat strongly inhibits Wg signaling by suppressing the effects of Arm.

### The AR2 domain is necessary for Arm function

**Figure 4:**
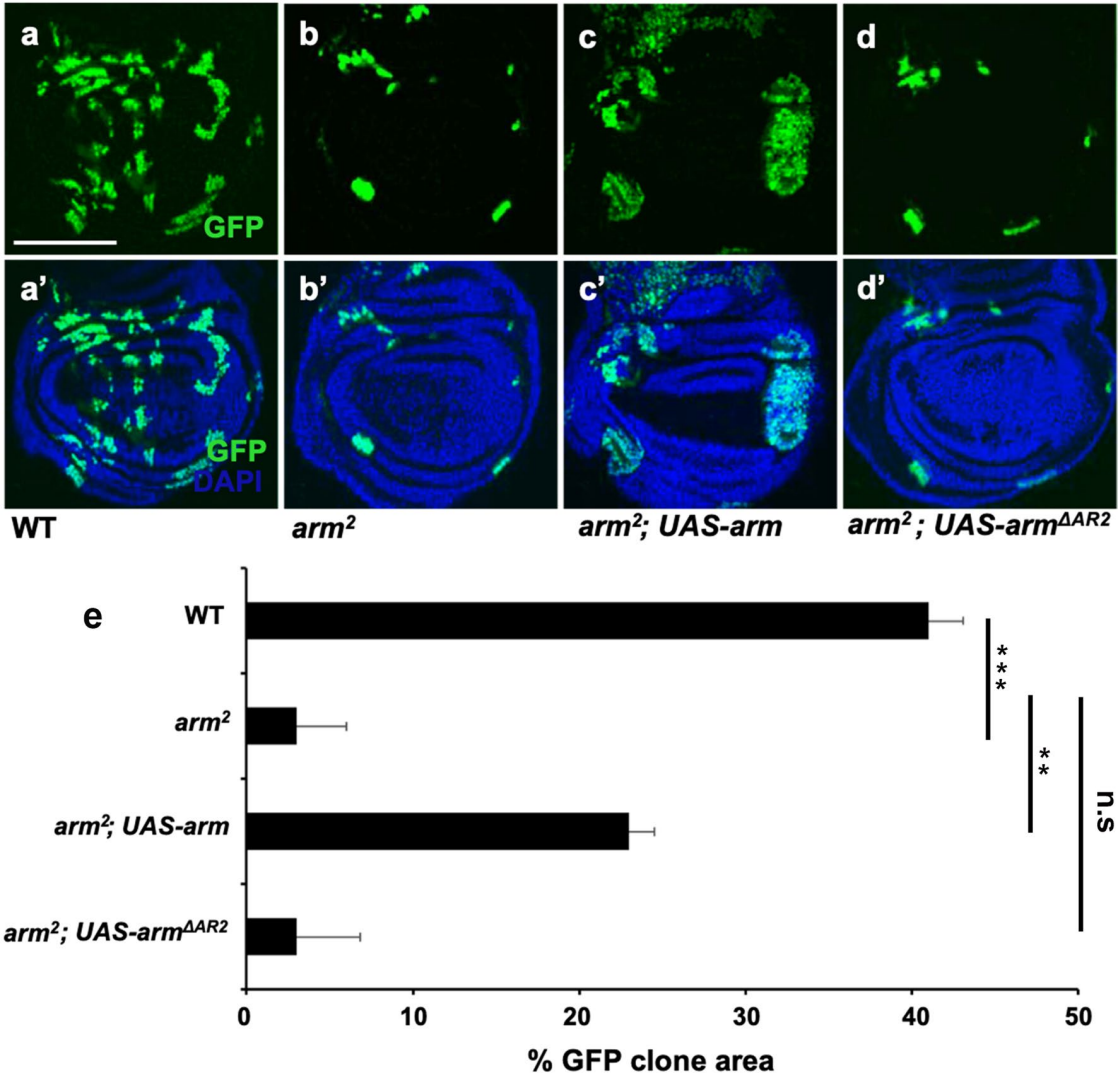
AR2 is essential for the function of Arm. MARCM strategy was used to induce Arm and Arm^ΔAR2^ expression in *arm*^*2*^ mutant clones in wing imaginal disc. Clones were monitored at 72 h after heat shock. Clones are marked by GFP in **a–d**. **a’–d’** show merge of GFP and DAPI. **(a–a’)** Wild-type clones. **(b–b’)** *arm*^*2*^ mutant clones. Clones are fewer and smaller. **(c–c’)** *arm*^*2*^ mutant clones with *UAS-Arm* expression are significantly larger in size. **(d–d’)** *arm*^*2*^ mutant clones with *UAS-Arm*^*ΔAR2*^ expression. *arm*^*2*^ mutant clones are not affected by Arm^ΔAR2^. **(e)** Quantification of clone sizes shown in **(a–d’)**. ’Percent GFP clone area’ on the X-axis indicates the fraction of the GFP-positive area in the entire disc (n = 10). Growth defects of *arm*^*2*^ mutant clones are suppressed by wild-type Arm but not by Arm^ΔAR2^.

Our data above suggested that AR2 overexpression is sufficient to interfere with the interaction between Arm and Klp64D, thus inhibiting Wg signaling. To determine whether the AR2 domain is necessary for the Arm function, we tested whether Arm protein lacking the AR2 domain can rescue the defects of loss-of-function *arm* mutation in developing wing discs. We induced *arm* mutant clones in 1st or 2nd instar larvae by using the MARCM method ^26^ and examined these clones in 3rd larval instar imaginal discs. Control wing discs showed many large wild-type GFP^+^ clones (Fig. 4a–a’). Compared with the wild-type control, homozygous *arm*^*2*^ null mutant clones were small and grew poorly in wing imaginal discs (Fig. 4b–b’, e). Overexpression of p35 in *arm*^*2*^ MARCM clones did not increase the clone size (Fig. S6). However, these *arm*^*2*^ mutant clones grew large when wild-type Arm was provided by *UAS-arm* within the clones (Fig. 4c–c’, e). On the contrary, *arm*^*2*^ mutant clones could not be suppressed by co-overexpression with Arm^ΔAR2^ that lacks the AR2 region (Fig. 4d–d’, e). Expression of Arm^ΔAR2^ was confirmed by Western blots of protein extracts from wing discs containing MARCM clones (Fig. S7). These data indicate that AR2 is essential for Arm function during wing development.

### Klp64D tail domain is essential for wing growth and its overexpression causes wing notching

**Figure 5:**
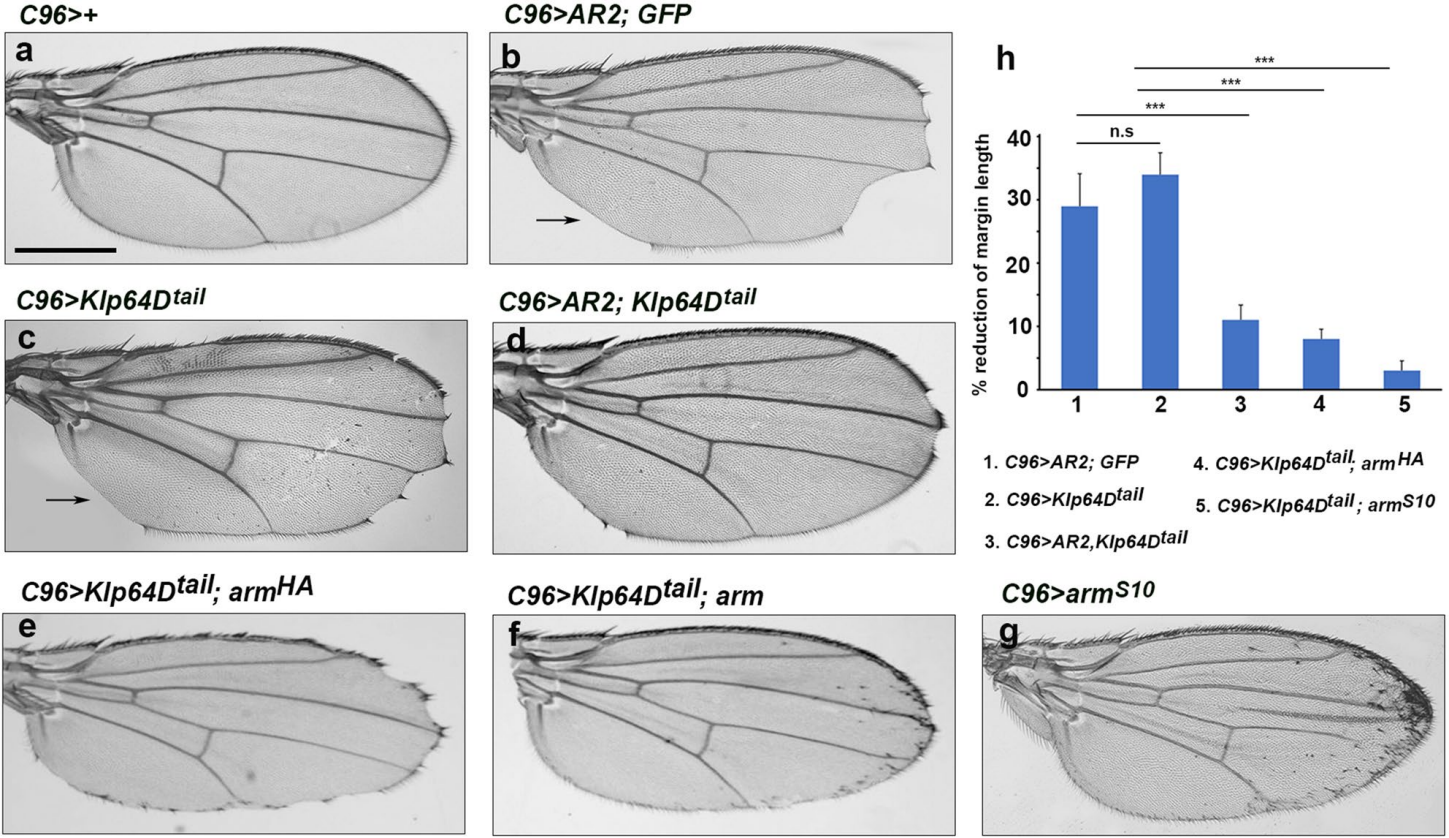
Klp64D tail domain is sufficient to inhibit the AR2 effects. **(a)** *C96* >  + wing as a control. **(b)** Overexpression of AR2 shows notching in the wing margin. **(c)** Overexpression of Klp64D^tail^ shows wing notching phenotype. **(d)** Overexpression of Klp64D^tail^ partially rescues the wing phenotype caused by AR2 overexpression. **(e)** Overexpression of wild-type Arm protein (Arm^HA^) partially rescues the notched wing phenotype of Klp64D^tail^ overexpression. **(f)** Overexpression of Arm^S10^ partially rescues the notched wing phenotype caused by Klp64D^tail^ overexpression. **(g)** Overexpression of Arm^S10^ induces extra wing margin bristles near the margin (arrows) but does not affect wing margin growth. **(h)** Quantification of notched wing phenotypes in **(c–g)**.

We have shown that the C-terminal tail domain of Klp64D is necessary and sufficient for binding to AR2 (Fig. 1e) and that AR2 overexpression results in notched wings (Fig. 2g). This AR2 phenotype was not affected by an additional copy of *UAS-GFP* (Fig. 5a, b). Interestingly, ubiquitous overexpression of Klp64D^tail^ by *act-Gal4* resulted in lethality during larval development. This suggested that like AR2, overexpression of the tail domain causes dominant-negative effects. Overexpression of Klp64D tail domain in wing disc using *C96-Gal4* induced frequent notching along the wing margin (32 ± 5% loss of wing margin, n = 30) (Fig. 5c, h). The notching by Klp64D^tail^ overexpression was probably not due to cell death since p35 failed to suppress the phenotype (Fig. S4). Furthermore, the wing notching phenotype by AR2 (Fig. 5b) was strongly suppressed by Klp64D^tail^ overexpression to only 11 ± 2% loss of margin (n = 30) (Fig. 5d, h). These data suggest that overexpressed AR2 and Klp64D^tail^ can titrate each other by direct binding, thus reducing their dominant-negative effects.

To check whether the dominant effects of Klp64D tail domain are due to an inhibition of Arm function, we co-expressed Arm in the DV boundary by *C96-Gal4*. Overexpression of wild-type Arm (Arm-HA) partially suppressed the notching phenotype of Klp64D^tail^ overexpression, showing only 7 ± 3% (n = 30) reduction in the margin (Fig. 5e, h). Activated Arm (Arm^S10^) resulted in slightly stronger suppression to 3 ± 2% margin loss (n = 30), respectively (Fig. 5f, h). Overexpression of Arm^S10^ alone induced ectopic wing margin bristles but did not significantly increase wing growth (Fig. 5g, h). These data suggest that overexpression of Klp64D tail dominantly inhibits the function of Arm.

**Figure 6:**
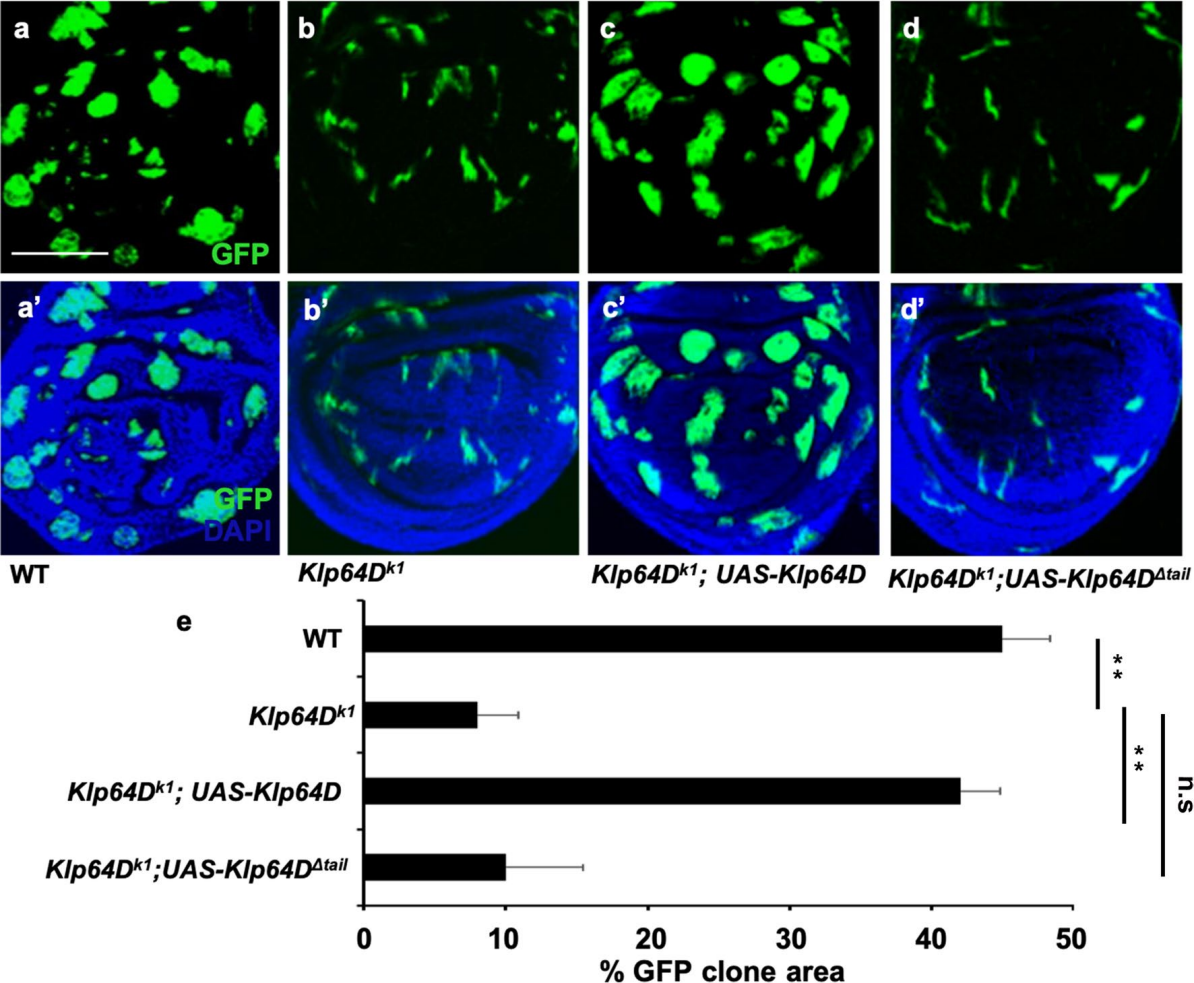
The tail domain is essential for the function of Klp64D. MARCM was used to induce Klp64D or Klp64D^Δtail^ expression in *Klp64D*^*k1*^ mutant clones in wing imaginal disc. Clones are marked by GFP in **(a–d)**. **(a’–d’)** show merges of GFP and DAPI channels. **(A–A’)** Wild-type clones. **(B–B’)** *Klp64D*^*k1*^ mutant clones are small. **(c–c’)** *Klp64D*^*k1*^ mutant clones with *UAS-Klp64D* expression are significantly larger than *Klp64D*^*k1*^ clones. **(d–d’)** *Klp64D*^*k1*^ mutant clones are not rescued by Klp64D^Δtail^. **(e)** Quantification of clone sizes shown in **(a–d’)**. ‘Percent GFP clone area’ indicates the fraction of the GFP-positive area in the entire disc (n = 10). Growth defects of *Klp64D*^*k1*^ mutant clones are suppressed by wild-type Klp64D but not by Klp64D^Δtail^.

We then tested whether the Klp64D tail domain is required for the Klp64D function, using mutant clones generated by MARCM. *Klp64D*^*k1*^ lethal hypomorphic mutant clones (GFP^+^) were very small compared with the size of wild-type control clones (Fig. 6a, a’), confirming the requirement of Klp64D for growth of wing tissue (Fig. 6b, b’, e). Like the *arm*^*2*^ mutant clones, *Klp64D*^*k1*^ MARCM mutant clones were not rescued by p35 expression (Fig. S6), *arm*^*2*^ mutant clones were small regardless of whether they are located close to the DV boundary or away from it (Fig. S6). This is expected because Arm is required cell-autonomously for Wg signal transduction. Similarly, *Klp64D*^*k1*^ mutant clones were small independent of their positions in wing pouch (Figs. 6 and S6). Homozygous *Klp64D*^*k1*^ mutant clones were rescued by expressing wild-type Klp64D, as shown by expanded growth of Klp64D-expressing clones (Fig. 6c, c’, e). However, *Klp64D*^*k1*^ mutant clones could not be rescued by overexpressing Klp64D^Δtail^, a mutant form deleted in the tail domain (Fig. 6d, d’, and e). Expression of mutated Klp64D^Δtail^ form was detected by Western blots of protein extracts from wing discs carrying MARCM clones (Fig. S7). Hence, the tail domain of Klp64D is required for the Klp64D function in wing growth, while its overexpression causes dominant-negative effects.

### Overexpression of AR2 or Klp64D tail causes increased localization of endogenous Arm with Golgi marker

**Figure 7:**
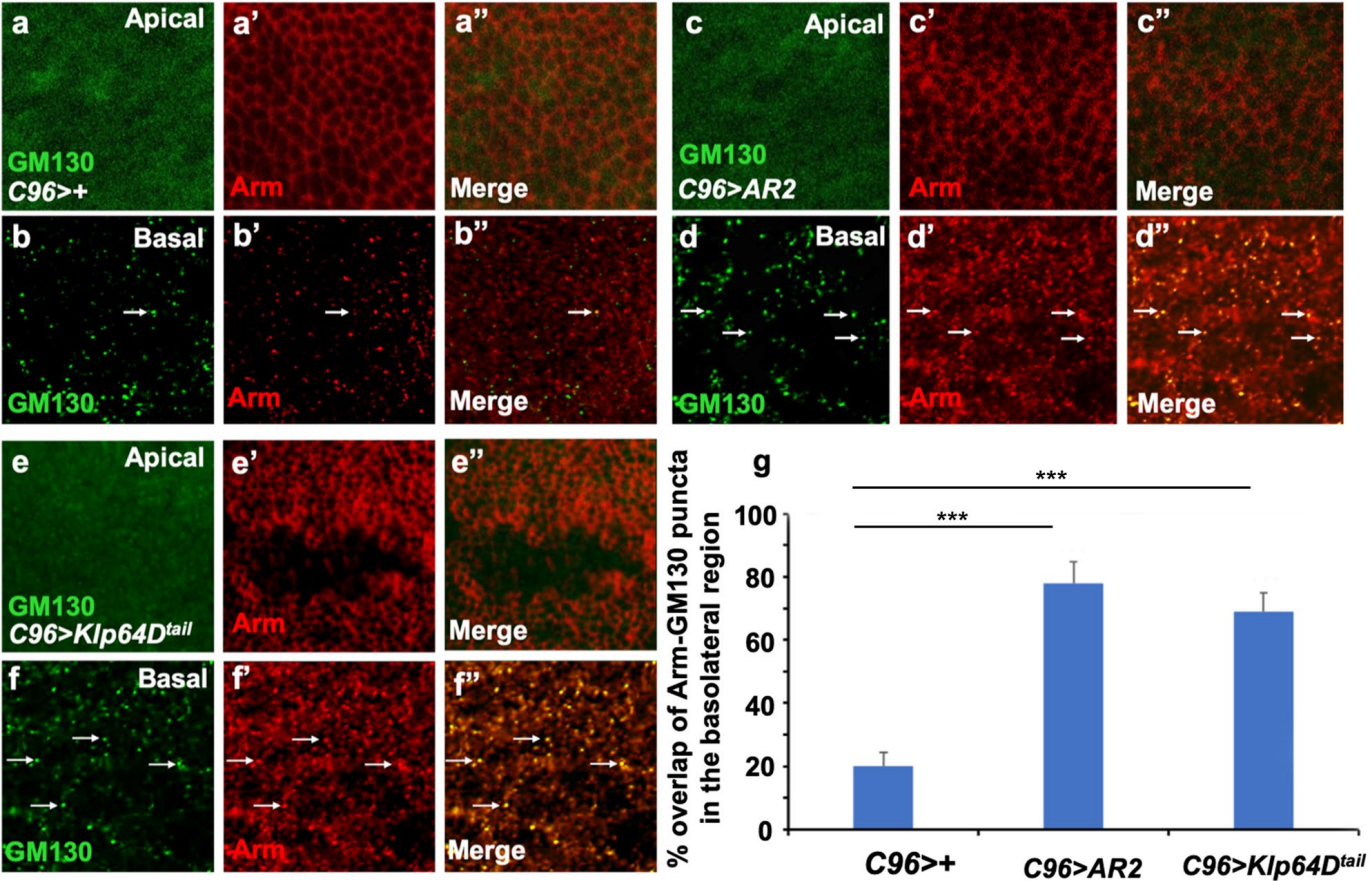
AR2 overexpression increases co-localization of Arm with Golgi vesicles. **(a–a”)** Apical section of *C96* >  + control wing disc. **(b–b”)** Basal section of *C96* >  + control wing disc. GM130 puncta (**b**, in green) overlap with Arm staining at a low level (< 20%) (**b’**, in red). Arrows in **(b**–**b”)** indicate representative overlaps between GM130 and Arm stains. **(a”)** is a merged image of **(a)** and **(a’)**. **(c–c”)** Apical section of *C96* > *AR2* control wing disc. **(d–d”)** Basal section of *C96* > *AR2* wing disc. It shows about 78% of punctate Arm overlapping with GM130 puncta. Arrows point to representative overlaps between GM130 and Arm stains in **(d–d”)**. **(d”)** is a merged image of **(d)** and **(d’)**. **(e–e”)** Apical section of *C96* > *Klp64D*^*tail*^ control wing disc. **(f–f”)** Basal section of *C96* > *Klp64D*^*tail*^ wing disc. It shows about 67% of punctate Arm overlapping with GM130 puncta. Arrows point to representative overlaps between GM130 and Arm stains in **(f–f”)**. **(f”)** is a merged image of **(f)** and **(f’)**. **(g)** Quantification of the overlap between Arm and GM130 puncta in *C96* >  + as a control, *C96* > *AR2* and *C96* > *Klp64D*^*tail*^ wing discs (n = 5).

It has been shown that depletion of Klp64D results in an accumulation of punctate Arm staining with Golgi vesicles in the basolateral region of wing disc cells^11^. Because AR2 overexpression causes dominant-negative effects on the Klp64D-Arm interaction, we examined whether AR2 overexpression also leads to increased overlap of Arm stain with Golgi. In wild-type control (*C96* > +), basolateral region of wing cells showed relatively low levels of GM130 vesicles that overlap with Arm puncta (20 ± 2% overlap, Fig. 7b–b”) whereas no GM130 vesicle was detected in the apical region (Fig. 7a). Overexpression of AR2 in the DV boundary region using *C96-Gal4* (*C96* > *AR2*) did not show significant effects on junctional Arm stains (Fig. 7a–a”; 7c-c”), but resulted in increased overlap of Arm puncta with Golgi vesicles in the basolateral region of DV boundary cells (78 ± 3% overlap, Fig. 7d–d”, 7 g). Similar phenotypes were found in the DV boundary region of wing disc depleted in Klp64D^11^. Overexpression of Klp64D^tail^ domain also increased the level of Arm puncta overlapping with GM130 in the basolateral region (67 ± 3% overlap, Fig. 7f–f”, g), while reducing the level of junctional Arm (Fig. 7e–e”). These results suggest that overexpression of AR2 or Klp64D^tail^ causes similar abnormal localization of Arm with Golgi in the basolateral region of DV boundary cells.

## Discussion

We have identified AR2 of Arm as a specific domain for interacting with the cargo domain of Klp64D motor protein. Overexpression of AR2 in the DV boundary region of wing disc causes consistent notching phenotype in the wing. Because Klp64D is required for Wg signal transduction by interacting with Arm^14^, the wing notching by AR2 overexpression might be due to dominant-negative effects of AR2 on Wg signal transduction. This is supported from the suppression or enhancement of AR2 wing notching phenotypes by overexpressing or silencing Klp64D, respectively. The severity of dominant-negative wing notching by AR2 overexpression was also correlated with the loss of Sens in the DV margin of wing disc.

AR2 overexpression strongly inhibits the Arm effects on Wg signaling activity in the TOP-Flash reporter assay, suggesting that AR2 overexpression antagonizes Arm function. Further, overexpression of the tail domain of Klp64D causes similar dominant notching effect along the wing margin. This suggests that Klp64D^tail^ competitively interferes with the normal interaction between Arm and Klp64D during wing margin development. This is also supported by the finding that the wing notching phenotype of AR2 overexpression is strongly suppressed by co-expression of Klp64D^tail^ (Fig. 5d). It should be noted that dominant-negative effects of AR2 or Klp64D^tail^ were tested by non-physiological condition of transgene overexpression. In addition, AR2 overexpression may also affect cell adhesion function of Arm at adherens junctions, leading to cell death. However, overexpression of AR2 or Klp64D^tail^ in wing disc does not cause apoptotic cell death (Fig. S4), and the notched wing phenotypes are not suppressed by p35. These data suggest that the effects of AR2 and Klp64D^tail^ are probably not due to non-specific cell death. We also found that AR2 overexpression did not significantly affect the junctional pattern of Arm staining in wing disc (Fig. 7c’), although AR2 may cause subtle cell adhesion defects that we could not detect. Interestingly, unlike AR2, overexpression of Klp64D^tail^ showed significant reduction in the junctional Arm staining, suggesting that it might affect both functions of Arm in cell adhesion and Wg signaling. However, the reduced level of junctional Arm did not result in noticeable increase in cell death (Fig. S4c’). To further overcome the limitation of our overexpression experiments, we also examined the loss of function analysis. Our data from MARCM clonal analysis suggest that the AR2 domain is essential for the function of Arm in wing development. Similar genetic tests also show that the tail domain is essential for the function of Klp64D in wing disc. Taken together, our data suggest that AR2 and Klp64D tail regions are important for the interaction between Arm and Klp64D and these domains are required for their normal function in wing margin development.

Proteins containing Arm repeats interact with their binding partners through a groove formed by several central Arm repeats. In *Drosophila* embryogenesis, the central most Arm repeats such as AR3-6 and 8 of Arm are important for Wg signal transduction^27^. However, other individual Arm repeats may also be involved in interaction with binding partners^28,29^. For example, AR1 region of β-catenin interacts with specific proteins such as the BCL9 transcriptional regulator^22^. AR12 region of Arm/$\beta$-catenin is involved in interacting with Hyrax/Parafibromin to activate Wg/Wnt target gene expression^30^. Biochemical and genetic interactions between AR2 and Klp64D also suggest a possible role of individual AR in interacting with specific partner proteins. The entire Arm repeat domain (aa 141–670) binds in vitro to two domains of Klp64D, the coiled-coil domain and the tail domain^11^. AR2 is the only repeat that interacts with the tail domain. Overexpressed AR2 may specifically interfere with the interaction between Arm and the cargo domain of Klp64D, leading to dominant-negative effects in the wing margin. In contrast, AR3, 7, 8, and 10 repeats can bind to Klp64D but not to its tail domain (Fig. 1d), suggesting that they may interact with the coiled-coil domain of Klp64D. Although AR3, 7, 8 and 10 do not show any dominant phenotypes when expressed in the DV boundary of wing disc, ubiquitous overexpression of AR2, 3, 7, or 8 results in larval lethality. Thus, AR3, 7, and 8 may also have specific dominant-negative effects during larval development, although the mechanism remains to be studied. It is worth noting that our binding assays between ARs and Klp64D were performed in vitro using GST fusion proteins. It would be interesting to test whether AR2 expressed as part of multiple AR repeats or intact Arm protein may be more stable and efficient for interacting with Klp64D. Ultimately, structural analysis will help understand how Klp64D^tail^ binds to the AR2 region of Arm.

It has been shown that Klp64D knockdown in the DV boundary region of wing disc results in increased overlapping localization of Arm with GM130 Golgi marker^11^. Our data also show that overexpression of AR2 or Klp64D tail region increases the number of endogenous Arm puncta co-localized with GM130 in the DV boundary region, consistent with the idea that AR2 overexpression may affect Klp64D function in Arm trafficking. Being a cytosolic protein, Arm is unlikely to be localized in the lumen of Golgi vesicles. However, cytosolic proteins can be bi-directionally transported between endosomes and Golgi^31^. Endosomal proteins also localize to endoplasmic reticulum (ER)^32^. Since Arm and Klp64D are co-localized with Rab5 endosome marker^11^, endosomes may be involved in Arm localization to Golgi. We also found that some Arm puncta overlap with DE-Cad (Fig. S8), suggesting that Arm may co-localize with DE-Cad on the way to the plasma membrane from ER. All three of DE-Cad, Arm and GM130 punctate stain were also found to overlap at a low level (Fig. S8). Precise mechanisms for the increased Arm localization with Golgi by Klp64D RNAi or AR2 overexpression needs further studies.

Arm and Klp64D are conserved orthologs of mammalian $\beta$-catenin and Kif3A, respectively. This raises a question whether the interaction between the Arm repeat AR2 and the tail domain of Klp64D may be also conserved. It is noteworthy that overexpression of human Kif3A can effectively rescue the wing notching phenotype caused by Klp64D RNAi^11^. Hence, it would be interesting to see whether the interaction between AR2 region of $\beta$-catenin and the cargo domain of Kif3A plays a role for the regulation of Wnt signaling in mammalian systems.

## Material and methods

### Genetics

All *Drosophila* strains were grown and maintained at room temperature (22 °C). *Klp64D*^*k1*^ allele causes a premature stop codon^10^ (stock number 5578, Bloomington Drosophila Stock center (BDSC), USA). UAS lines used were *UAS-Klp64D RNAi* (10642R-1 and 10642R-2 from National Institute of Genetics, Japan, v45373 from Vienna Drosophila Resource Center, Austria), *UAS-Klp64D*^33^, *UAS-arm RNAi* (8515 BDSC), *UAS-arm*^*S10*^ ^34^*, UAS-arm*, *arm*^*2*^ mutant (8369, 8554 BDSC) and *C96-Gal4* (BDSC).

### MARCM mutant clones

To generate *arm* mutant clones, males of *hs-FLP, FRT 19A tub-Gal80; nub-Gal4, UAS-GFP/UAS-GFP* were crossed with females *FRT19A arm*^*2*^*/FM7c* flies. Control clones were generated by crossing males of *hs-FLP, FRT19A, Tub-Gal80; nub-Gal4, UAS-GFP/UAS-GFP* with females of *FRT19A/FRT19A*. The same method was used to generate *Klp64D*^*k1*^ clones. Mutant clones were induced at 60 h after egg laying (AEL) by an hour heat shock at 37 °C. Larvae were aged at room temperature until 3rd instar (about 120 h AEL) and wing imaginal discs were dissected for immunostaining.

### Wing mounting

Wings from adult flies were dissected in isopropanol and mounted in Canadian Balsam mounting medium (Gary’s magic mount) following the protocol of Roberts and Lawrence^40^. Mounted wings were photographed under AxioImager Zeiss optics with 5 × and 10 × lenses. Alternatively, whole flies or portions of flies were photographed through a dissection microscope. Quantification of wing notching was performed using Image J software by selecting all the margin area and measuring the margin length that was lost by notching using the Measuring and Counting tool.

### Immunocytochemistry

Wing imaginal discs were dissected from third instar larvae. Wing discs were fixed in PBS/0.2% saponin on a rotator for at least 20 min at RT. Anti-Dlg antibody (gift from Kyung-Ok Cho), anti-Sens antibody (gift from Hugo Bellen), anti-Dll antibody and anti-GFP were diluted 1:500, 1:1,000, 1:500, and 1:200 respectively, in PBS/0.2% saponin/0.5% goat serum and incubated at 4ºC overnight (O/N), rinsed three times in PBS/0.2% saponin, incubated in anti-rabbit FITC (1:200), anti-mouse Cy3 (1:200) and in anti-pig Cy5 (1:200), anti-sheep FITC (1:200) for 2 h at RT, and rinsed again four times in PBS/0.2% saponin. Secondary antibodies conjugated with Cy3, Cy5 or FITC were from Alexa Flour (Molecular Probes). Fluorescent images were acquired using Zeiss confocal microscope.

### Transgene construction

To generate transgenic flies, Arm repeat domains (AR2, AR3, AR7, AR8, AR10), Arm-HA, Arm deleted in the AR2 domain (Arm^∆AR2^) and Klp64D tail domain (Klp64D^tail^) were amplified by PCR using DGRC LD23131 cDNA (for Arm) and genomic fly extract (for Klp64D) and cloned into a *pUAS-attB* vector (VK1, second chromosome 2R 59D3)^35^ using NotI and XbaI sites. The following primers were used to make Arm repeats constructs:

AR2: 5′-GCGGCCGCCAAAATGGGAGATGGAGGGAGATCCACT-3′ and 5′-GCTCTAGACATACCGGTGTCCAGGTCGAA-3’.

AR3: 5′-GCGGCCGCCAAAATGTTCCCGCAGAATTTCACACAA-3′ and 5′-GC TCTAGACATTTCATATCGTCCACTTGGTCT-3’.

AR7: 5′-GCGGCCGCCAAAATGGGAACCGTCACAAATGCTAAA-3′ and 5′-GC TCTAGACATTTGACCACCGCGTGCTTTAGC-3’.

AR8: 5′-GCGGCCGCCAAAATGTCTGATCAACTACCAGGACGA-3′ and 5′-GC TCTAGACATTGGCCCTGGTTGCCAGCTCAG-3’.

AR10: 5′-GCGGCCGCCAAAATGTTCCCAGGCCGCCATGATGGT-3′ and 5′-GC TCTAGACATTGTTCATAATGGCATGTCGCG-3’.

### Transient transfection of S2R + cells

S2R + cells were grown in M3 media (Sigma) supplemented with 10% 10X insect medium supplement (Sigma) and 1% penicillin–streptomycin solution. Cultured cells were transfected with 1–3 µg DNA using Cellfectin II Reagent following a protocol from the manufacturer (Thermo Fisher Scientific).

### Cell culture transfections for Topflash assay

*Drosophila* S2R + cells express all of the Wg signaling components necessary to respond to exogenously added Wg^36^. For Arm, *UAS-ArmAR2* and *USA-HA* control transfections, 1 µg DNA, 1 µg WISER (luciferase reporter plasmid) (gift from J.P Vincent) and 1 µg *act-Gal4* (in the well of *UAS-HA*) in 98 µl EC buffer were combined with 16 µl Enhancer (Qiagen), 38 µl Effectene (Qiagen) in 3.5 × 10^6^ cells/1.6 mL of growth media in a 6-well plate. To assess the effect of Arm and Arm AR2 on Wg signaling, 400 µl of media with or without Wg protein was added 2 days after transfection. 1 day later, the cells were lysed to assess luciferase levels using the Dual-Luciferase Reporter Assay System (Promega) containing Tcf reporter (firefly luciferase) and a constitutively active control reporter (renilla luciferase). Wg activity was calculated as the ratio of firefly to renilla luciferase activities with SEM (Standard error of the mean) of three independent experiments with the HA control. Statistical significance was based on a two-tailed *t* test. To obtain Wg-containing media, S2 *tub-wg* cells (DGRC) and S2 cells were grown in M3 media (Sigma) as described^37^. Cells were pelleted by centrifugation. The presence of Wg protein was confirmed by Western blotting by mouse anti-Wg antibody (DSHB).

### In vitro binding assays

For GST pull down, the following recombinant proteins were expressed and purified: GST-Klp64D^FL^, GST-Klp64D^C^, GST-Klp64D^N^, GST-Klp64D^Tail^, MBP-Arm^FL^, MBP-Arm repeats, MBP-Arm^C^, MBP-Arm^N^ or MBP-AR domains^38^. Arm repeats used were: AR1 (aa 149–188), AR2 (aa 189–231), AR3 (aa 232–272), AR4 (aa 273–314), AR5 (aa 316–357), AR6 (aa 358–398), AR7 (aa 399–437), AR8 (aa 438–481), AR9 (aa 482–527), AR10 (aa 528–595), AR11 (aa 596–636), and AR12 (aa 637–677). Bacterial cell lysates were prepared as described^39^. An equal amount of blocked glutathione Sepharose 4B beads (Bioprogen) with GST, GST fusion protein or beads alone were incubated with lysates containing MBP-fusion proteins O/N at 4ºC. After several washes with pull-down buffer (20 mM Tris pH 7.5, 150 mM NaCl, 0.5 mM EDTA, 10% glycerol, 0.1% Triton X 100, 1 mM DTT, and protease inhibitor cocktail), sample buffer was added, beads were boiled and protein were resolved on SDS-PAGE. For western blotting, proteins were electrophoretically transferred onto nitrocellulose membranes, blocked in 5% skim milk (Biorad) and incubated with primary goat anti-GST (Santa Cruz) or rabbit anti-MBP antibody (Santa Cruz), and secondary goat anti rabbit or anti goat (Molecular Probes) antibody. Protein bands were visualized using ECL kit (GenDepot).

## Supplementary information

Supplementary Information.
