# Supplementary material for: Role of Armadillo repeat 2 and kinesin-II motor subunit Klp64D for wingless signaling in Drosophila
Source: Sci Rep. 2020 Aug 17;10:13864. doi: 10.1038/s41598-020-70759-8 (PMC7431425; doi:10.1038/s41598-020-70759-8)
Supplement: Supplementary file 1 — Supplementary Information. [file 41598_2020_70759_MOESM1_ESM.pdf]

Role of Armadillo repeat 2 and kinesin-II motor subunit Klp64D for Wingless signaling in *Drosophila*

Linh Thuong Vuong[1,2], Jong-Hoon Won[1], Minh Binh Nguyen[1,2] and Kwang-Wook Choi[1*]

[1] Department of Biological Sciences, Korea Advanced Institute of Science & Technology, Daejeon-305-701, Korea

[2] Previous address: Department of Cell, Developmental and Regenerative Biology, Icahn School of Medicine at Mount Sinai, New York, New York 10029, USA

[*]Correspondence: kchoi100@kaist.ac.kr

SUPPLEMENTAL INFORMATION

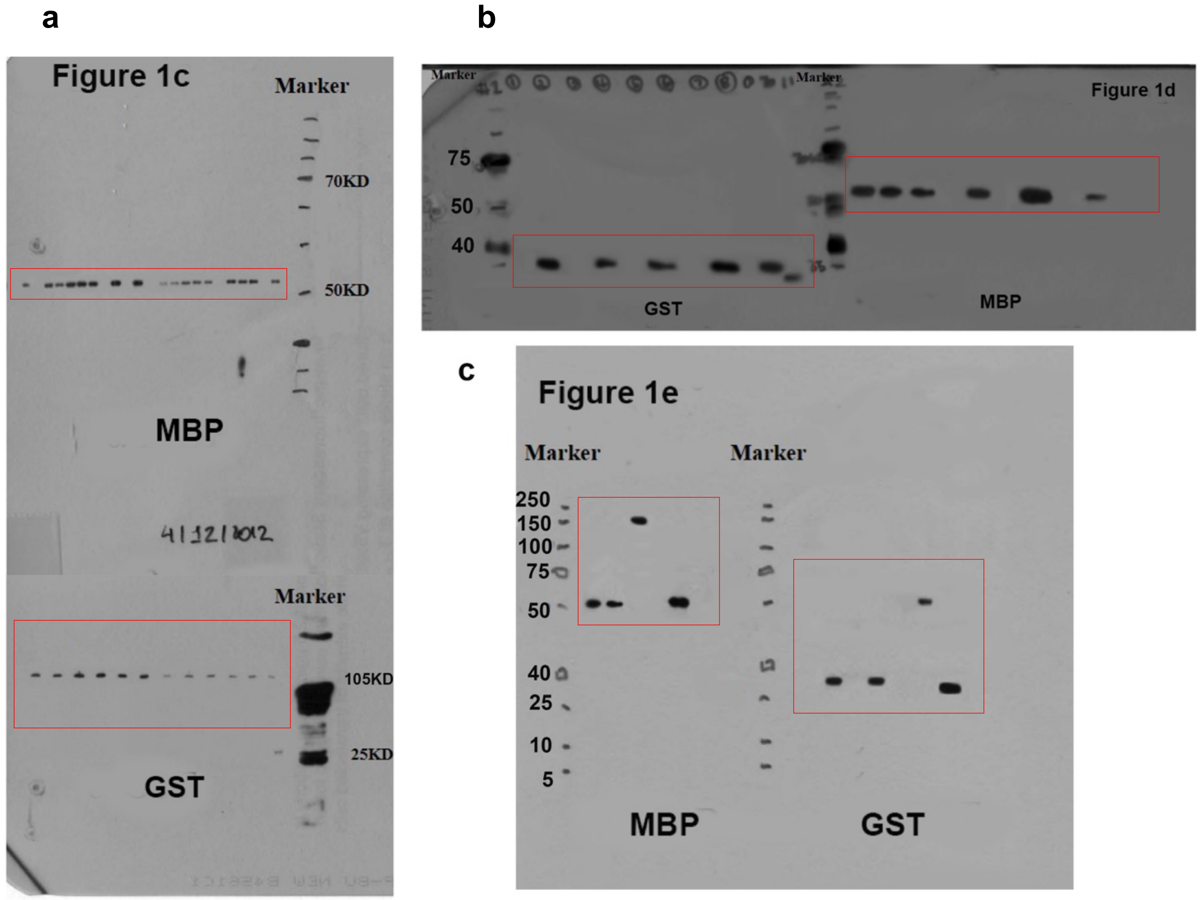

Figure S1 (related to Figure 1). Full length autoradiograms of all Western blots in Figure 1

(a-c) Original blots of the pull-down assays between AR domains of Arm and Klp64D. (a) Uncropped autoradiogram of Figure 1c shows pull-down results between twelve individual AR domains of Arm and GST-Klp64D^{FL}. (b) Full length blots of Figure 1d shows five AR domains of Arm (AR2, 3, 7, 8, 10) were pulled down by GST-Klp64D^{tail}. (c) The original autoradiogram of Figure 1e shows direct binding of Klp64D^{tail} and AR2. Cropped areas are indicated by red boxes.

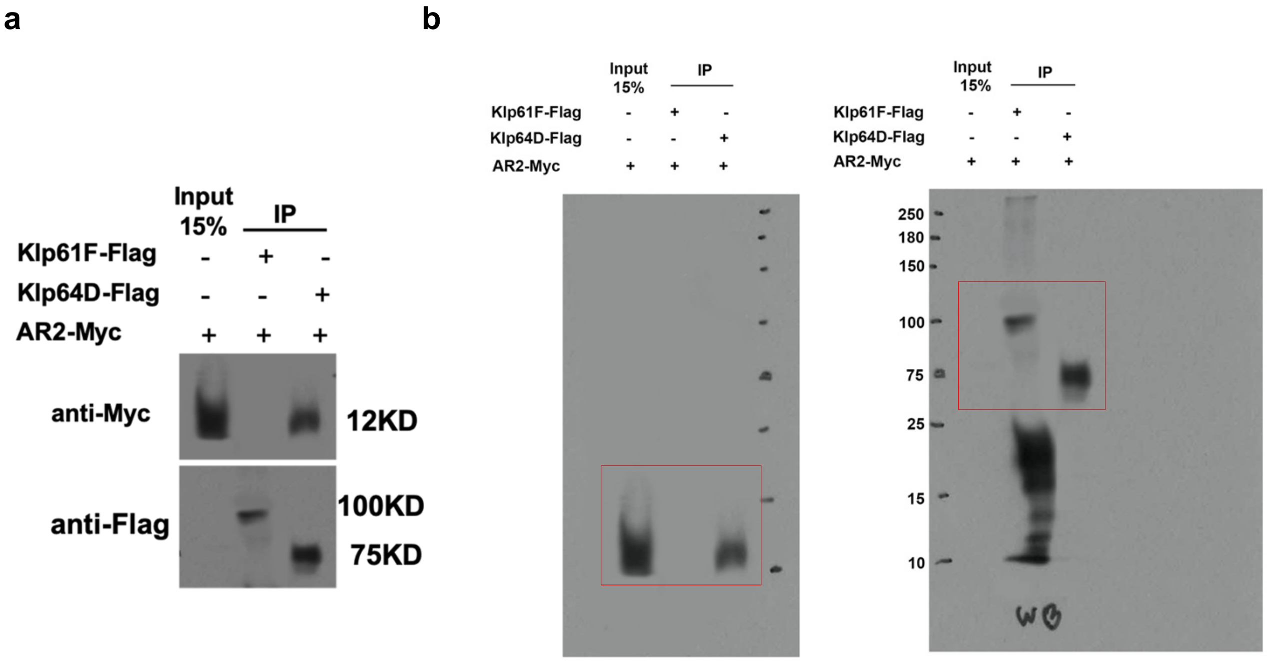

Figure S2 (related to Figure 1). Co-immunoprecipitation between AR2 and Klp64D in *Drosophila* S2 cell and full-length autoradiograms

(a) Co-immunoprecipitation of Klp64D and Arm. S2 cells were transfected with Myc-AR2 (input 15%, lane 1) or co-transfected with Flag-Klp61F and Myc-AR2 (lane 2) or Flag-Klp64D and Myc-AR2 (lane 3). Cell lysate was immunoprecipitated by anti-Flag antibody-conjugated beads and was analyzed by western blotting.

(b) Original autoradiogram of the co-immunoprecipitation assay between AR2 and Klp64D. Cropped areas are indicated by red boxes.

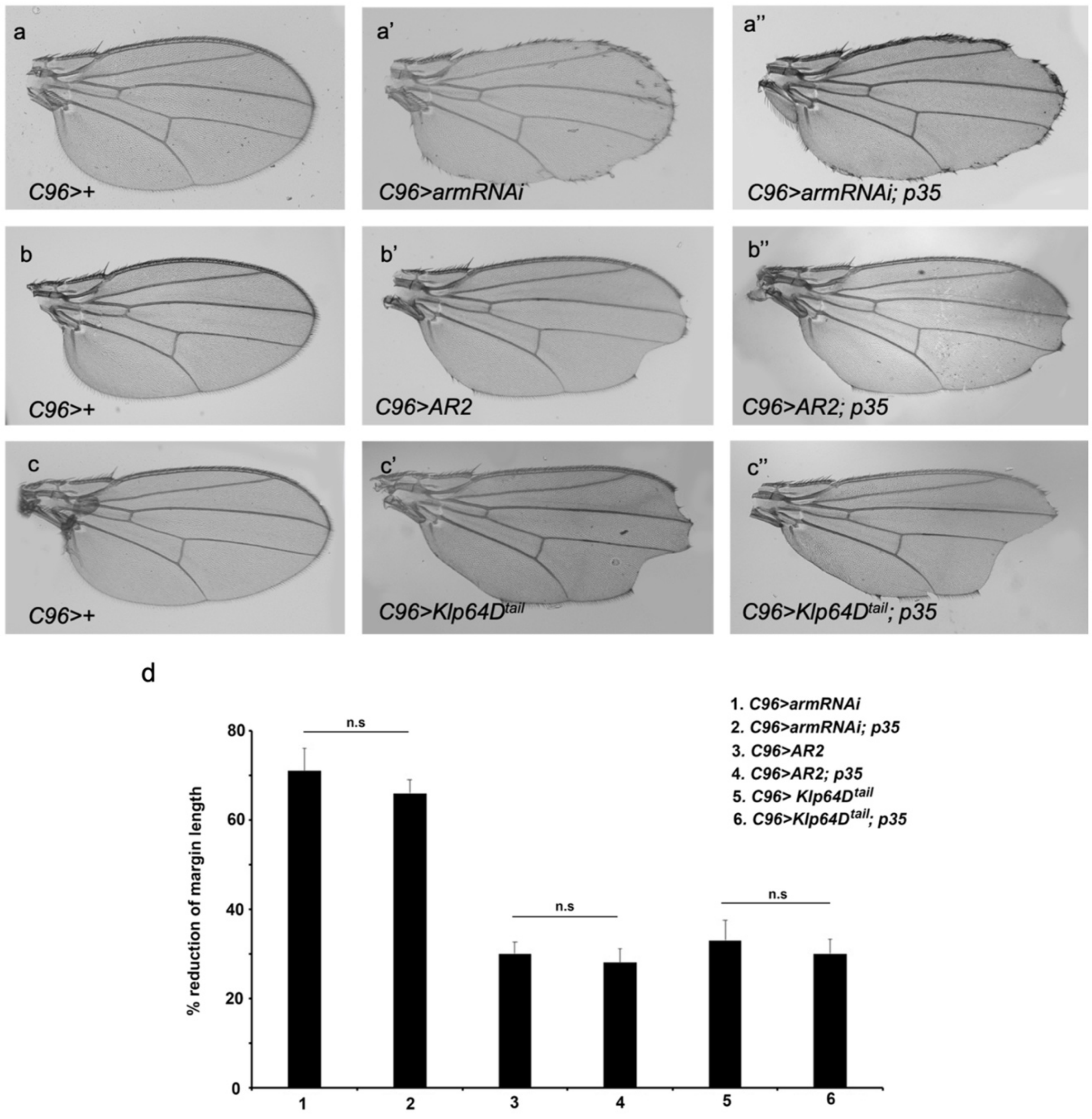

Figure S3 (related to Figure 2). Wing notching by overexpressing AR2 or Klp64D^{tail} overexpression is not due to cell death.

(a) *C96-Gal4*>+ as a control. (a') *arm RNAi* causes notched wings. (a'') Overexpression of p35 does not rescue the notched wing phenotype.

(b-b'') Wing notching by AR2 overexpression (b') is not suppressed by p35 (b''). (c-c'') Wing notching by Klp64D^{tail} overexpression (c') is not suppressed by p35 (c'').

(d) Quantification of notched wing phenotypes shown in (a-c'')

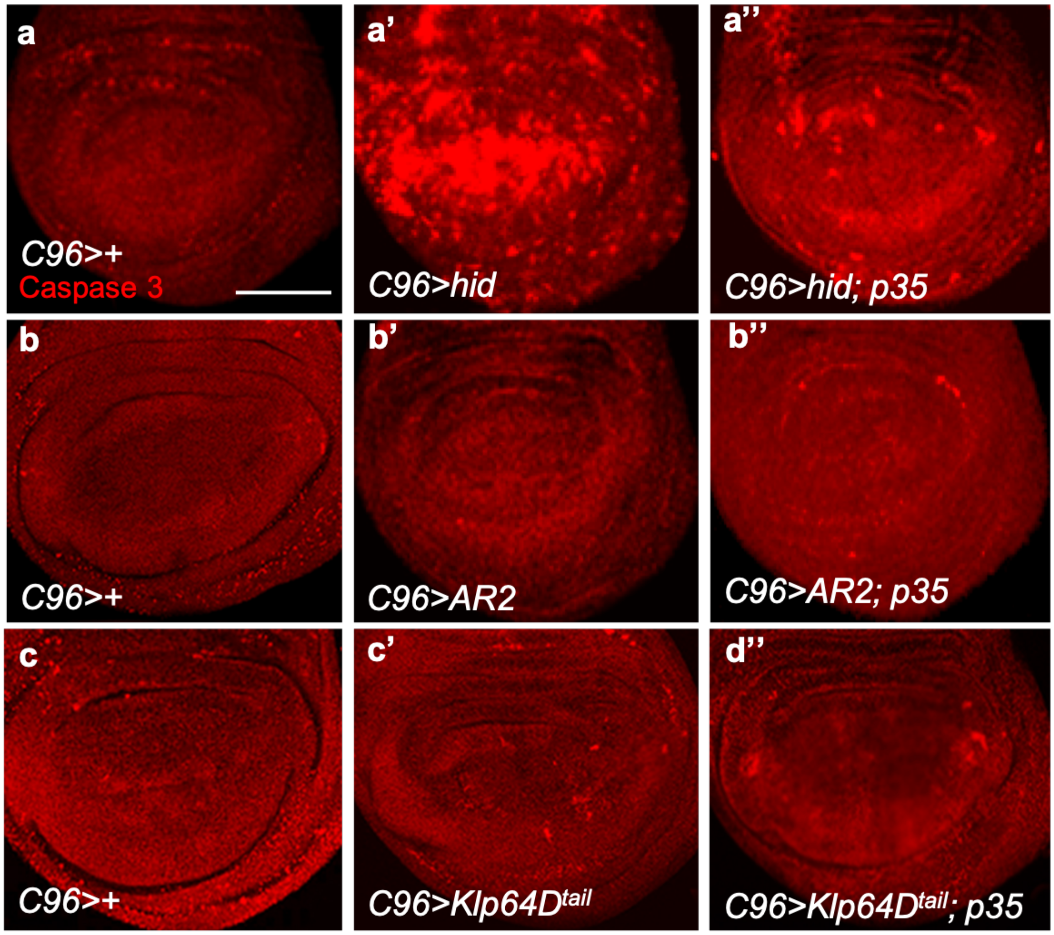

Figure S4 (related to Figure 2). Overexpression of ArmAR2 or Klp64D^{tail} does not induce cell death.

(a-a”) Hid overexpression by *C96-Gal4* induces caspase activation marked by cleaved Cas3 staining (a’). Cell death by Hid is suppressed by p35 overexpression (a”).

(b-b”) AR2 overexpression does not induce cell death (b’). p35 overexpression has no effect on Cas3 staining (b”).

(c-c”) *Klp64D^{tail}* overexpression does not induce cell death (c’). p35 overexpression has no effect on Cas3 staining (c”).

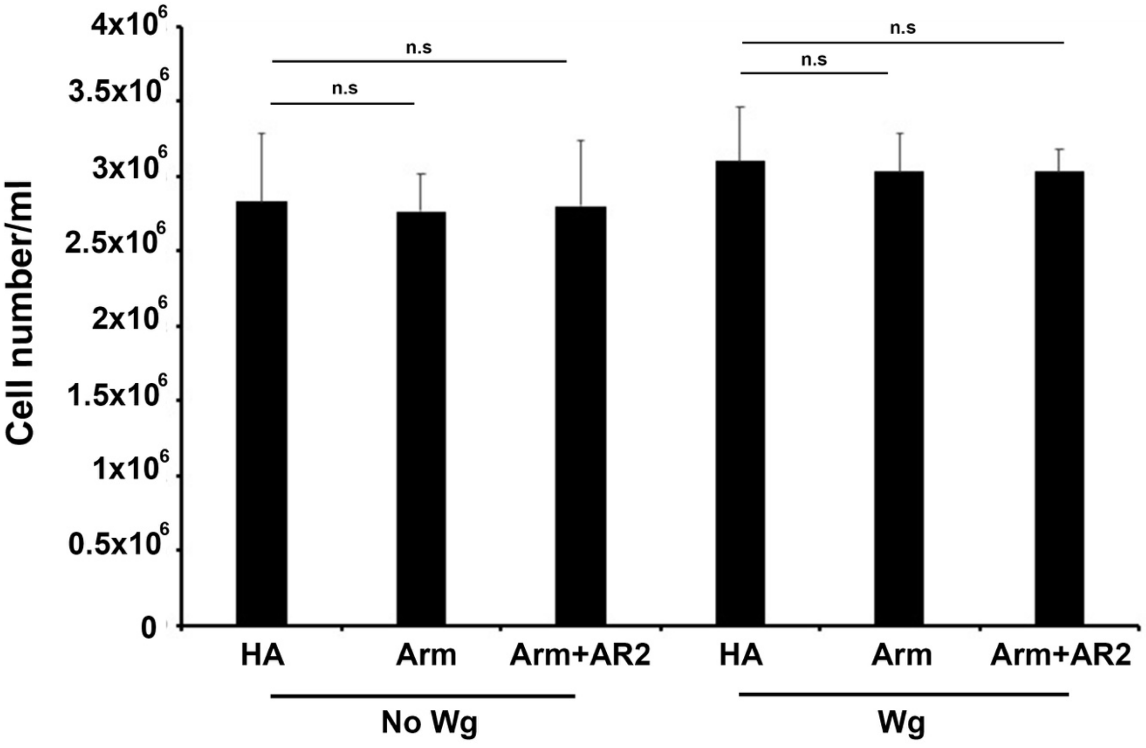

Figure S5 (related to Figure 3). Effects of AR2 overexpression on S2 cell survival
S2 cells were cultured for luciferase assay for Wg signaling activity. Cell numbers were not significantly affected by addition of Wg-expressing culture media or expression of Arm and AR2.

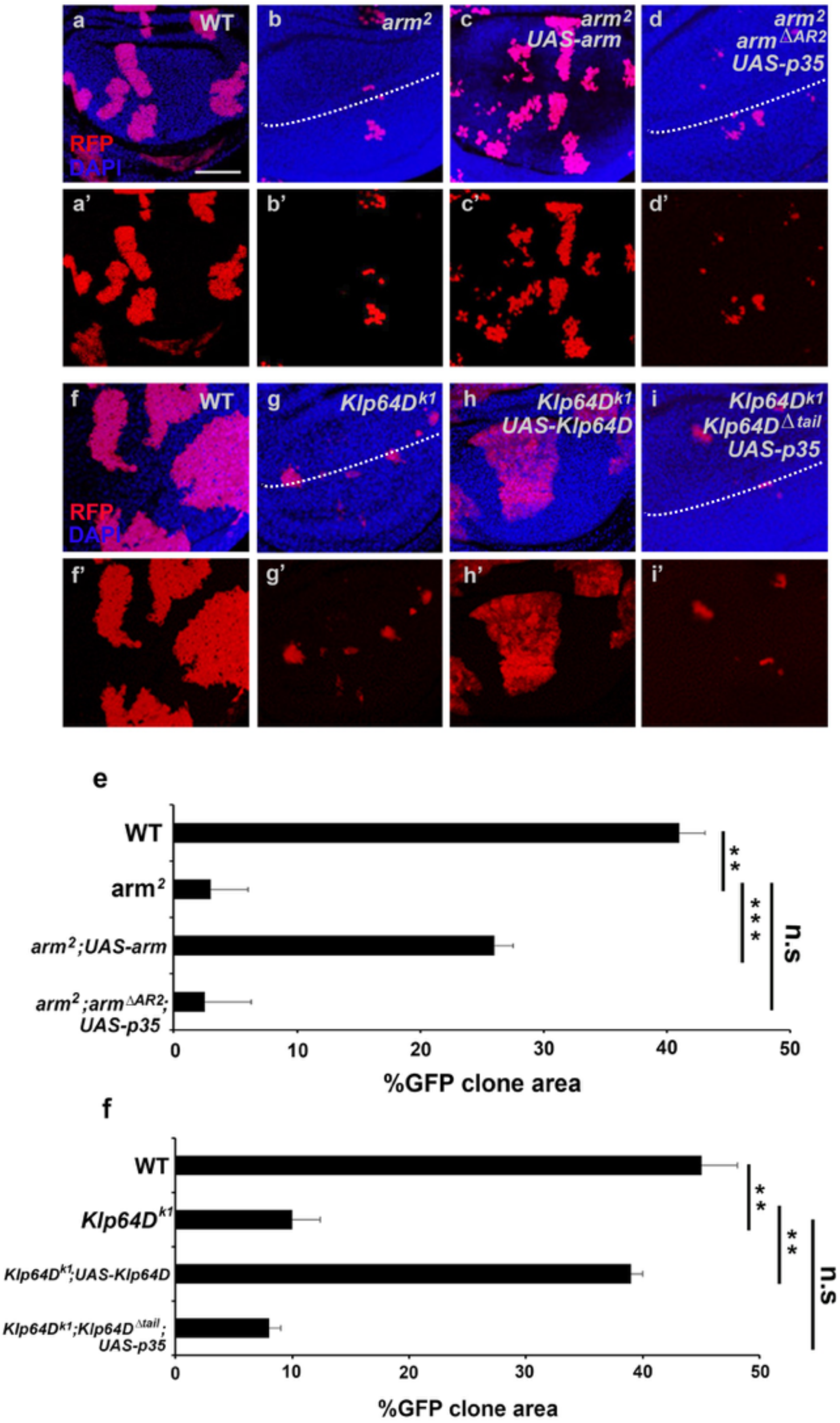
a
WT
RFP
DAPI
b
arm2
c
arm2
UAS-arm
d
arm2
armΔAR2
UAS-p35
a'
b'
c'
d'
f
WT
RFP
DAPI
g
Klp64Dk1
h
Klp64Dk1
UAS-Klp64D
i
Klp64Dk1
Klp64DΔtail
UAS-p35
f'
g'
h'
i'
e
WT
arm2
arm2;UAS-arm
arm2;armΔAR2;
UAS-p35
**
***
n.s
0
10
20
30
40
50
%GFP clone area
f
WT
Klp64Dk1
Klp64Dk1;UAS-Klp64D
Klp64Dk1;Klp64DΔtail;
UAS-p35
**
**
n.s
0
10
20
30
40
50
%GFP clone area

Figure S6 (related to Figures 4 to 6). AR2 and Klp64D^{tail} are essential for the function of Arm and Klp64D, respectively.

(a-d) Wing discs containing MARCM clones are stained by DAPI. Clones are marked by RFP. Genotypes are indicated in the panels (a-d). *arm*^{2} mutant clones are small (b) compared with wild-type control clones (a). *arm*^{2} mutant clones are restored by overexpression of wild-type Arm (c). *arm*^{2} mutant clones are not rescued by co-expression of *arm*$^{\Delta AR2}$ and p35 (d). (a'-d') RFP channel images of (a-d). (e) Clone sizes in (a-d) are quantified. 'Percent GFP clone area' on the X-axis indicates the fraction of the RFP-positive area in the entire disc (n=5).

(f-i) *Klp64D*^{k1} mutant clones are small (g) compared with wild-type control clones (f). *Klp64D*^{k1} mutant clones are restored by overexpression of wild-type Klp64D (h). *Klp64D*^{k1} mutant clones are not rescued by co-expression of *Klp64D*$^{\Delta tail}$ and p35 (i). (f'-i') RFP channel images of (a-d). (j) Clone sizes in (f-i) are quantified. 'Percent GFP clone area' on the X-axis indicates the fraction of the RFP-positive area in the entire disc (n=5). Dashed lines in (b), (d), (g), (i) indicate DV boundary.

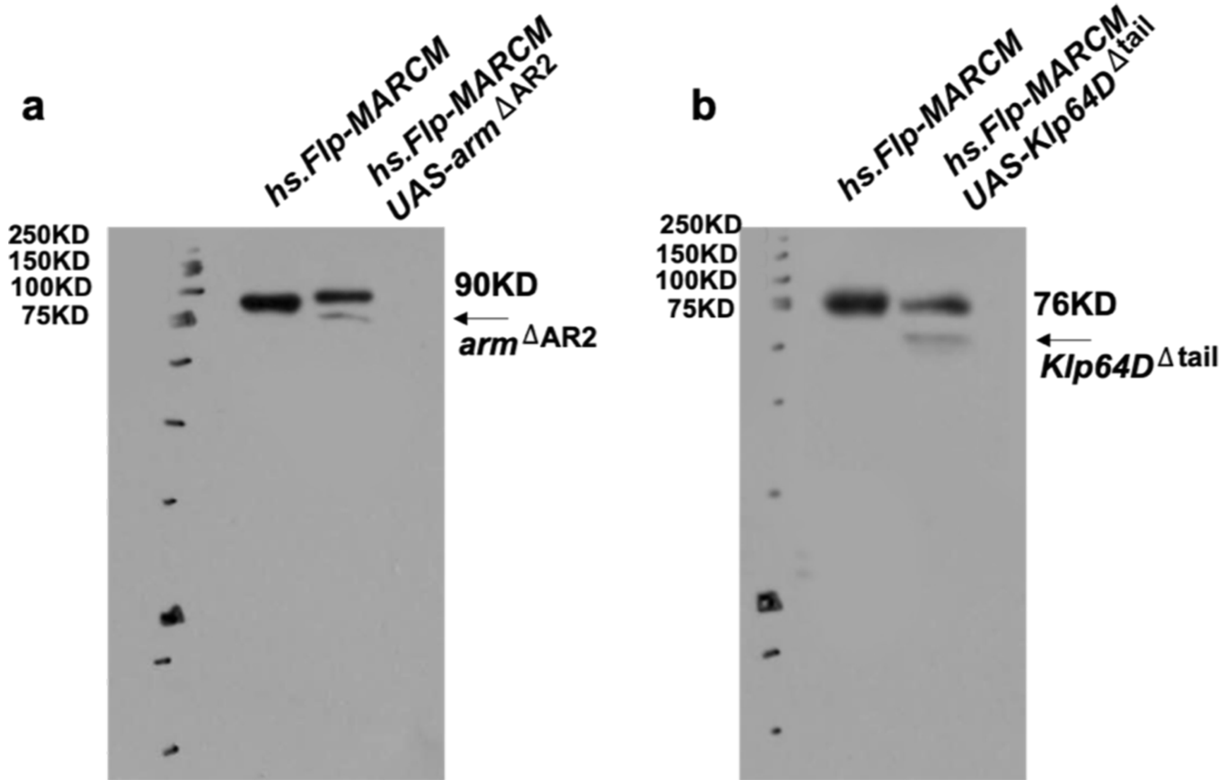

Figure S7 (related to Figures 4 and 6). Detection of Arm$^{\Delta AR2}$ and Klp64D$^{\Delta tail}$expression. (a) *arm^{2}* MARCM clones expressing Arm were generated by *hs-flp.* 10 pairs of wing discs containing these clones were collected and homogenized in 50μl of 20X SDS sample buffer. Expression of Arm and Arm$^{\Delta AR2}$ (arrow) were detected by anti-Arm immunostaining of western blot. Wing discs containing control wild-type MARCM clones were used as loading control. (b) *Klp64D^{k1}* MARCM clones expressing Arm were generated by *hs-flp*. Protein extracts were prepared as in (a). Expression of Klp64D and Klp64D$^{\Delta tail}$ (arrow) were detected by anti-Klp64D. Wing discs containing control wild-type MARCM clones were used as loading control.

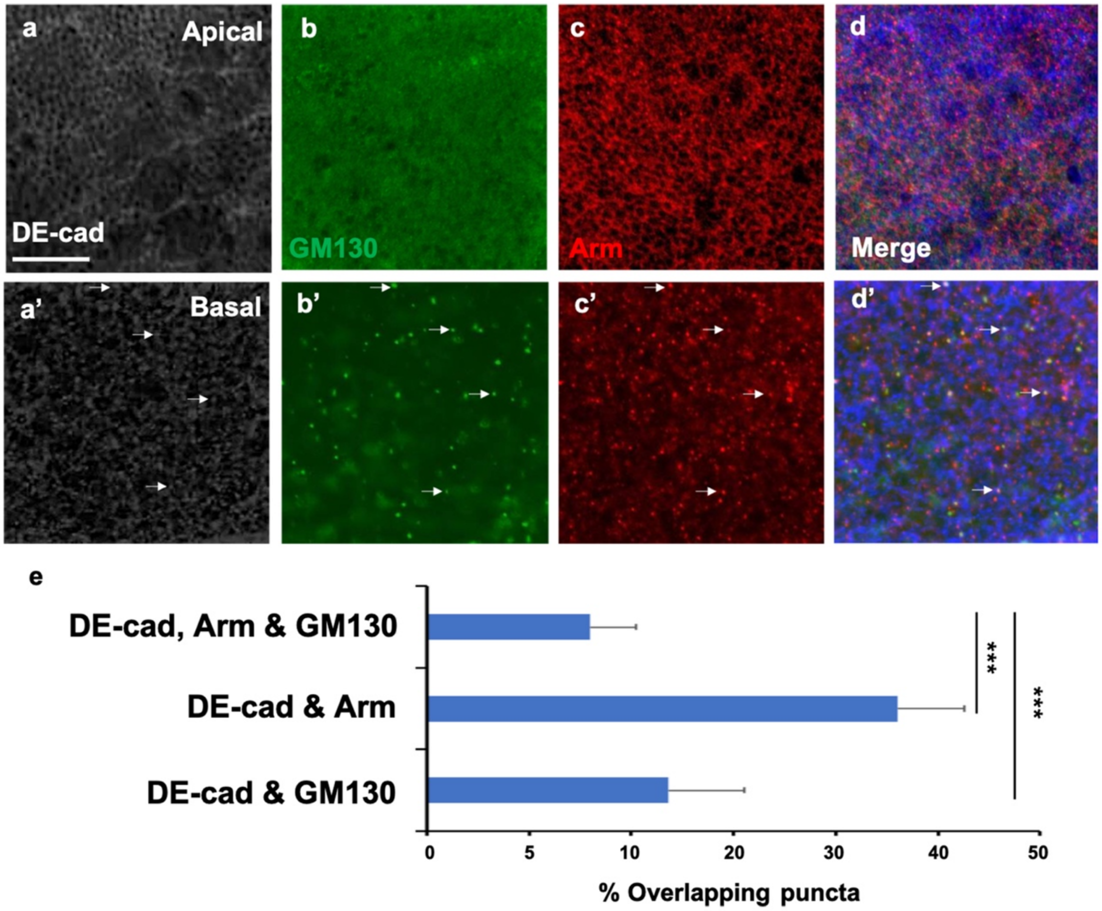

Figure S8 (related to Figure 7). Co-localization between GM130, DE-cad, and Arm. (a-d) Apical section of *C96*>+ wing disc. (a'-d') Basal section of *C96*>+ wing disc. DE-cad puncta (a', in white) overlap with GM130 (b', in green) and Arm (c', in red) staining at low level (<10%). (e) Quantification of overlapping puncta between De-cad, Arm and GM130.
